# The application of quality improvement concepts, strategies, and tools to enhance participation in clinical trials among Latino families

**DOI:** 10.1017/cts.2024.557

**Published:** 2024-10-10

**Authors:** Keanaan Malke, Jennifer R. Hemler, Daniel Lima, Pablo Colon, Caroline Mendoza, Naomy Azcona, Katie A. Devine, Thomas I. Mackie, Usha Ramachandran, Darlene Forbes, Michael Lucas, Shawna V. Hudson, Manuel E. Jimenez

**Affiliations:** 1 Rutgers New Jersey Medical School, Newark, NJ, USA; 2 Department of Family Medicine and Community Health, Rutgers Robert Wood Johnson Medical School, New Brunswick, NJ, USA; 3 The Boggs Center on Developmental Disabilities, Department of Pediatrics, Rutgers Robert Wood Johnson Medical School, New Brunswick, NJ, USA; 4 Rutgers Cancer Institute of New Jersey, New Brunswick, NJ, USA; 5 Department of Pediatrics, Rutgers Robert Wood Johnson Medical School, New Brunswick, NJ, USA; 6 Department of Health Policy and Management, School of Public Health, SUNY Downstate Health Sciences University, Brooklyn, NY, USA; 7 Department of Pediatrics, Eric B. Chandler Health Center, New Brunswick, NJ, USA; 8 Department of Pediatrics, Central Jersey Medical Center, Perth Amboy, NJ, USA; 9 Saint Peters University Hospital Pediatric Faculty Group, New Brunswick, NJ, USA; 10 Children’s Specialized Hospital, New Brunswick, NJ, USA

**Keywords:** Latinos, recruitment, quality improvement, clinical trials, disparities

## Abstract

Underrepresentation of people from racial and ethnic minoritized groups in clinical trials threatens external validity of clinical and translational science, diminishes uptake of innovations into practice, and restricts access to the potential benefits of participation. Despite efforts to increase diversity in clinical trials, children and adults from Latino backgrounds remain underrepresented. Quality improvement concepts, strategies, and tools demonstrate promise in enhancing recruitment and enrollment in clinical trials. To demonstrate this promise, we draw upon our team’s experience conducting a randomized clinical trial that tests three behavioral interventions designed to promote equity in language and social-emotional skill acquisition among Latino parent–infant dyads from under-resourced communities. The recruitment activities took place during the COVID-19 pandemic, which intensified the need for responsive strategies and procedures. We used the Model for Improvement to achieve our recruitment goals. Across study stages, we engaged strategies such as (1) intentional team formation, (2) participatory approaches to setting goals, monitoring achievement, selecting change strategies, and (3) small iterative tests that informed additional efforts. These strategies helped our team overcome several barriers. These strategies may help other researchers apply quality improvement tools to increase participation in clinical and translational research among people from minoritized groups.

## Introduction

People from Latino backgrounds are now the second largest ethnic group in the USA [1]. In 2021, children from Latino backgrounds represented 26% of US children [2] compared to 14% in 1993 [3]. Despite these trends, between 2007 and 2020, the median proportion of Latino children participating in clinical trials was just 7%, compared to 66% for White children [4].

Efforts to increase participation in clinical trials among people from minoritized groups and enhance reporting span decades. For example, the Revitalization Act of 1993 sought to ensure that National Institutes of Health (NIH)-funded research included data on gender and race/ethnicity and examined differential effects between groups [5]. In 2016, the 21^st^ Century Cures Act [6] required applicable clinical trials to submit results of valid analyses by gender, race, and ethnicity to ClinicalTrials.gov. More recently, the National Institute on Minority Health and Health Disparities strategic plan included major goals focused on enhancing diversity and inclusion in NIH-funded research and clinical trials [7]. Despite existing efforts, reporting remains inconsistent, and representation of people from minoritized groups in clinical trials is not reflective of recent demographic shifts [8]. In an analysis of 20,000 studies from 2000 to 2020, 43% of studies reported race/ethnicity data, and among those that did, people from racial and ethnic minoritized groups remained underrepresented [9]. Another study analyzed gender and race representation among novel cardiometabolic drug approval trials from 2008 to 2017. Among 125 trials that reported race, 81% of participants identified as White as compared to 4% who identified as Black, and among trials reporting ethnicity, only 11% identified as Latino. The total number of women enrolled in all the trials comprised 36%, with no significant increase over time [10].

Previous studies have identified barriers that are complex, multifactorial, and may limit participation in clinical trials among people from Latino backgrounds specifically. These barriers include mistrust, lack of information, time and resource constraints, language barriers, low literacy, fear of losing healthcare benefits, and risks associated with immigration status [8,11–14]. However, to date, knowledge of these barriers has not translated into consistent action that could increase participation, for example, language and literacy accommodations [14].

Clinical trials drive advances in knowledge and treatment. Underrepresentation of people from racial and ethnic minoritized groups in clinical trials diminishes their external validity, limits uptake of innovations, and restricts access to the potential benefits of participation [15]. In this article, we describe how we applied quality improvement concepts, strategies, and tools derived from the Model for Improvement [16] to successfully recruit Latino parent–infant dyads for a randomized clinical trial, “Addressing Disparities in Language and Social-emotional Skill Acquisition through Literacy Promotion in Primary Care: Literacy Promotion for Latinos Study.” The study took place during the COVID-19 pandemic, which intensified the need for responsive recruitment procedures and materials [17]. We describe the concepts, strategies, and tools we employed throughout the recruitment process and include case examples to illustrate how they can enhance participation among people from minoritized groups in research.

## Study context

### Literacy promotion for Latinos study

This ongoing clinical trial (ClinicalTrials.gov Identifier NCT04609553) tests the extent to which outreach text messages and referrals to enhance access to poverty-reducing resources, in combination with standard literacy promotion, enhances child language and social-emotional skill acquisition among Latino children from under-resourced communities. This clinical trial builds on our team’s prior community-engaged research [18–21] that informed interventions and outcomes assessed. Specifically, local community leaders and Latino parents identified early childhood development and school readiness as prioritized goals and identified pediatric professionals as being well positioned to address these goals [18,19]. The interventions respond to barriers identified by Latino parents who participated in the pilot work. Specifically, they highlighted the need to provide reminders to engage in parenting routines like shared reading and address economic hardship that interferes with parents’ ability to engage in activities like shared reading [20,21]. Moreover, the study assesses outcomes that parents identified as important including parenting, language development, and social-emotional skills.

### Participants and setting

We recruited 630 Latino parent–infant dyads from three community health centers (CHCs) in Middlesex County, New Jersey (N.J.) (Table 1) [22]. New Brunswick and Perth Amboy, where the CHCs are located, have large Latino populations (46% and 78%), with sizeable proportions born outside the USA (77% and 91%) [23–25]. Two CHCs have similar annual pediatric patient volumes (∼ 5,500 pediatric patients/year), with 73% and 66% identifying as Latino, respectively. The third has higher annual pediatric patient volume (∼20,000 pediatric patients/year) and 55% identify as Latino. Many of these CHCs’ patients prefer receiving health information in Spanish. Inclusion criteria were primary caregiver of a child 6–12 months, Latino background, primary language English or Spanish, cellphone ownership, age≥18 years, willing to receive text messages, and willing to accept randomization. Exclusion criteria were children with genetic disorders or previously identified developmental delays, inability to provide informed consent, and intent to discontinue care at recruitment sites.

**Table 1.** Characteristics of caregivers (*N* = 630)

Caregiver born in US or US Territories	
Yes	65 (10%)
No	565 (90%)
**If not born in any of the U.S. states, years lived here**	9 (6.9)
**Latino ethnicity**	630 (100%)
**Country of origin**	
Mexico	230 (37%)
Dominican Republic	132 (21%)
Honduras	104 (17%)
Other^1^	143 (25%)
**Preferred language**	
English	74 (12%)
Spanish	556 (88%)
**English language proficiency**	
Speaks very well	77 (12%)
Speaks well	109 (17%)
Speaks not well	230 (37%)
Speaks not at all	212 (34%)
Refused	2 (0.3%)

1 Other country of origin: Argentina, Brazil, Chile, Columbia, Costa Rica, Ecuador, El Salvador, Guatemala, Nicaragua, Peru, Panama, Puerto Rico, and Uruguay.

## The Model for Improvement

### Overview

We incorporated quality improvement concepts, strategies, and tools from the Model for Improvement to enhance our recruitment procedures. The Associates for Process Improvement developed the Model for Improvement to improve processes and outcomes in diverse contexts, and it has been used widely in healthcare settings [16]. The model consists of two major components: (1) addressing three fundamental questions (What are we trying to accomplish?; How will we know a change is an improvement?; and, What changes can we make that will result in improvement?) and (2) implementing Plan-Do-Study-Act (PDSA) cycles to test changes. The model offers extensive strategies and tools to accelerate improvements [16]. Below, we describe how we addressed these three fundamental questions. We then provide illustrative examples of tests of change describing the factors that inhibited recruitment and the ways we used the Model for Improvement tools to help us overcome these barriers and reach our recruitment goals.

### Forming the team

The interdisciplinary study team was formed over several research projects. Most members had longstanding relationships with each other, the CHCs, and the local community. The principal investigator (PI)’s research focuses on promoting health equity for Latino communities; as such, he had partnered with community members and the CHCs on other projects. The core study team meets at least weekly and includes the PI, research assistants (RAs), two research coordinators, and a data manager. The RAs conducting recruitment and enrollment were all bilingual; most had a familial connection to Spanish, were based in the area, and had connections to the university and/or community. One research coordinator who has experience running a family medicine residency research network oversaw CHC and clinician engagement. The other research coordinator, who had worked on the preliminary studies and other community-based projects, oversaw participant engagement and RA training. In addition to providing oversight of surveys and outreach techniques, he helped the RAs write a handbook outlining protocols and scripts. All RAs used this handbook during training and reviewed it regularly. Training included several mock outreach calls and study visits with real-time feedback. RAs shadowed the research coordinator during actual study visits; the coordinator then shadowed at least three of each RA’s outreach calls and study visits, which were followed by debriefing sessions. Once RAs demonstrated competency, they conducted visits independently. For the remainder of the study, RAs met one-on-one with the research coordinator weekly to review cases for quality assurance and met one-on-one with the PI at least monthly with more frequent contact via phone, text, and email.

We identified clinician champions at the three CHCs who served as important team members. For one site, two clinicians joined the research team as content experts on literacy promotion and community health, in addition to their expertise on day-to-day clinic operations. At the other two CHCs, one clinician served as study champion. The clinician champions raised awareness about the study, helped develop recruitment protocols, and met monthly with the study coordinator who oversaw CHC engagement to provide updates and feedback.

The remaining team members included experts in implementation science, health services, social science, linguistics, and statistics. Multiple members had run successful clinical trials. An advisory panel of members of community and professional organizations provided feedback at least annually.

### Setting goals, establishing measures, and selecting changes

The initial proposal, which was developed before the pandemic, called for recruiting 630 parent–infant dyads in 2 years, which would require 6 enrollments/week. It took 2 years and 7 months to achieve this goal.

At the beginning of the study, we selected run charts to conduct time trend analysis and monitor progress toward our recruitment goals tracking the weekly total of clinician referrals, enrolled participants, and individuals who declined participation. We set up regular team meetings and check-ins with the CHCs to identify problems and brainstorm potential solutions in a participatory manner.

**Figure 1.**
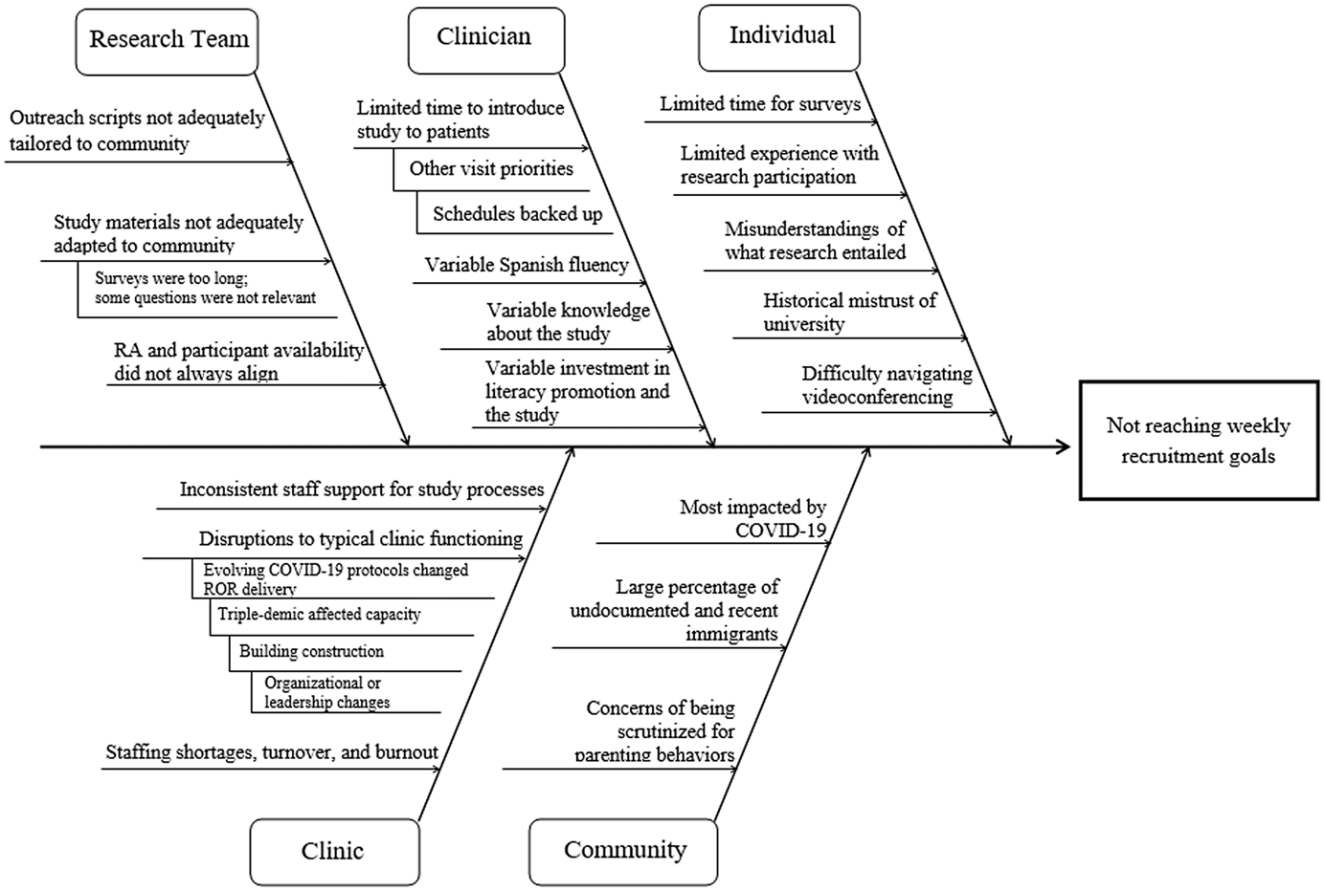
Cause and effect diagram of factors related to not reaching weekly recruitment goals.

During weekly core team meetings, RAs presented the run charts, shared their observations, discussed barriers they encountered, and brainstormed potential solutions. For example, RAs observed that certain terms caused confusion (see case example “Describing the study in culturally appropriate language” below). The study coordinator overseeing CHC engagement, and an RA also met monthly with clinician champions to assess factors that influenced processes and referrals. These meetings helped the team understand patient volume changes, clinician scheduling changes, and turnover. The coordinator and RA kept detailed fieldnotes and reported updates to the PI and core team during weekly meetings. As described below, the PI eventually delivered quarterly report-backs to each CHC via Zoom on recruitment and study status to enhance clinician engagement and answer questions and to elicit recruitment challenges directly from the clinicians on the ground. Figure 1 summarizes the challenges we encountered in a cause and effect diagram [16]. Cause and effect diagrams, also known as fishbone diagrams, visually outline how issues at different levels contribute to an underlying problem, in our case, not meeting our weekly recruitment goals.

Based on the recruitment data and meetings, the team agreed on changes collaboratively. Information and opinions were gathered from participants during informal debriefs after study visits, the CHC champions, and from our other team members.

### Testing and implementing changes

PDSA cycles were used to make modifications to the study processes. During weekly team meetings, the team would identify potential solutions to problems discussed, which would then be tested to determine if they were effective. In the following section, we provide specific examples. Application of QI tools allowed for data-driven improvements in study recruitment, reductions of participant burden, and integration of culturally appropriate language to facilitate the recruitment initiatives.

## Case examples

### Adapting recruitment strategies to a virtual environment

We recruited parent–infant dyads from November 2020 to June 2023. Originally, we planned for bilingual RAs to conduct in-person recruitment in the three CHCs. However, this was not feasible at the start of the study due to physical distancing and safety guidelines. In response, we developed a virtual recruitment plan in consultation with clinician champions at the three CHCs. The protocol called for clinicians to introduce the study; study staff offered guidance on how to briefly introduce the study to parents and refer them to the study team if interested. Referrals were sent electronically to the study team using secure, encrypted email. Bilingual RAs called parents to provide information and screen for eligibility; they then set up a secure videoconference (i.e., Zoom) to conduct the informed consent process and enrollment. While most participants enrolled on Zoom, some enrolled by phone. The most common reasons were difficulty setting up the application and connecting to the meeting.

### Increasing referrals from clinicians

**Figure 2.**
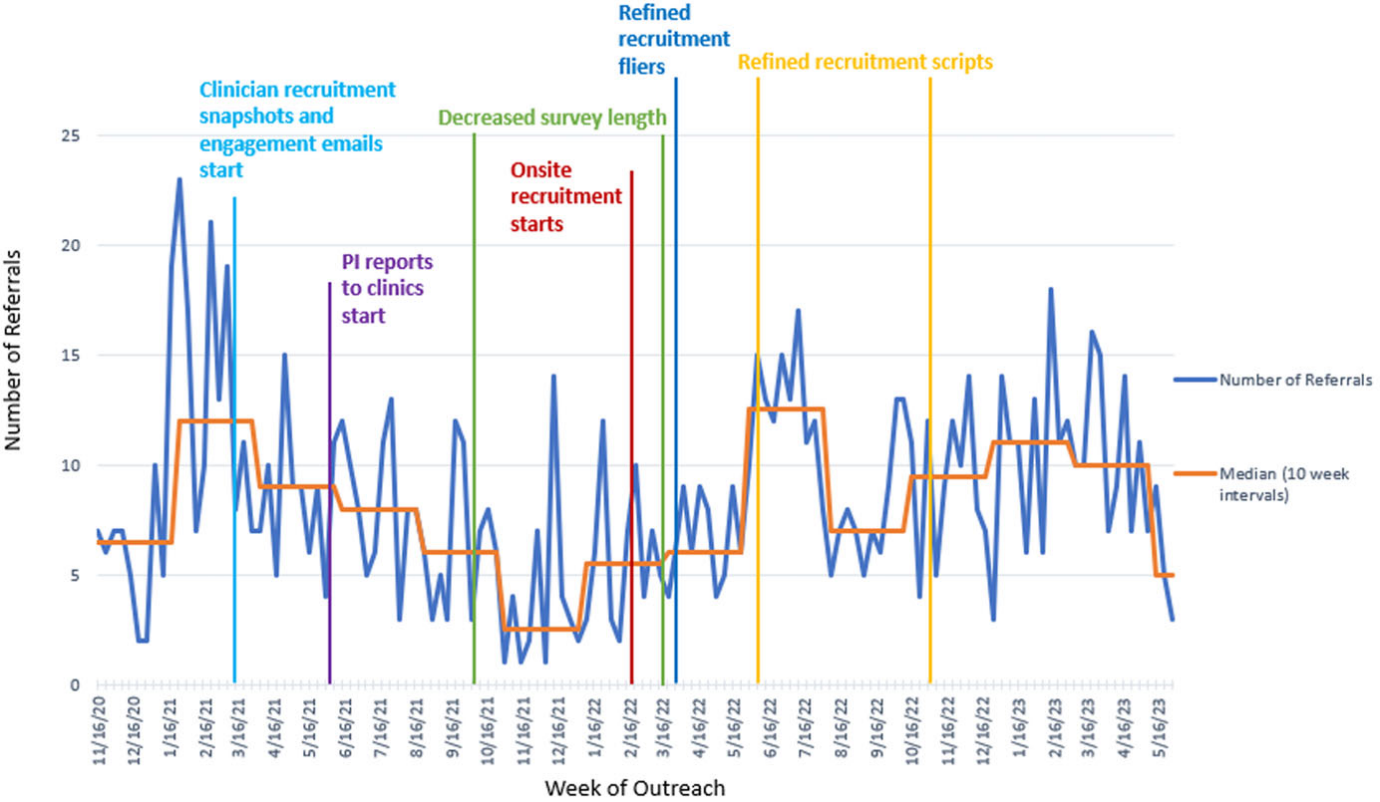
Total weekly referrals.

The team piloted the virtual recruitment procedures in October 2020. Despite the pilot, we encountered challenges receiving referrals from clinicians. Figure 2 illustrates referral volumes throughout the study. After the initial spike in clinician referrals at the beginning of the study, the referrals decreased. Only certain clinicians were sending in referrals consistently and at lower rates than those needed to achieve 6 enrollments/week. Based on our previous work, we anticipated approximately 60% of individuals referred to the study would participate. Based on this estimate, we needed 10 referrals per week on average. However, by week 50 (November 1, 2021) the median number of referrals was just 2.5/week (Figure 2). The team decided that changes were needed to enhance clinician and staff engagement and tested multiple measures iteratively. These included monthly emails with recruitment statistics for each CHC and celebratory emails for holidays or events (e.g., Latino Heritage Month) that included a recruitment tip or note about the local community (e.g., the local library offers free library cards). One of the research coordinators worked directly with a CHC staff member who agreed to remind clinicians to make referrals. The PI also began to provide quarterly report-backs directly to the CHC clinicians. The frequency was identified collaboratively with the clinician champions to enhance engagement, but not increase clinician participation burden. While the research and clinical teams perceived that these changes enhanced rapport and communication, they did not have a sustained effect on increasing the number of referrals (Figure 2).

### Reducing perceived participation burden to enhance recruitment

Parents who declined to participate frequently cited limited time as the reason. Informal feedback from participants also suggested the enrollment surveys were too long (>1 hour). During meetings, the team discussed which survey questions were redundant or unnecessary. We then made changes to reduce the number of surveys and questions and tested both timing and quality of participant experience. Additionally, participants were given the choice of completing the surveys in more than one call, so that we did not take up too much of their time at once.

**Figure 3.**
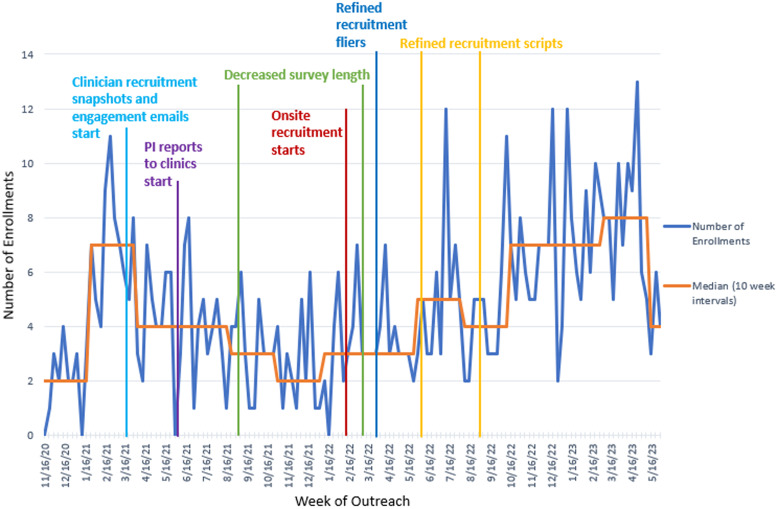
Total weekly enrollments.

The RAs conducted a series of time trials in a PDSA cycle to assess survey length and determine the longest duration acceptable to participants. They noted points at which participants seemed to lose engagement. Additionally, with more practice, the RAs gained greater confidence and efficiency. These efforts resulted in decreasing the median administration time from 80 minutes from January to February 2021, to 54 minutes from October to November 2021, and finally to 41 minutes in May 2022. RAs were able to tell prospective participants that the surveys would take under an hour to complete. These changes corresponded with a gradual increase in the percentage of individuals who agreed to participate, with the median number of weekly enrollments peaking at 8 per week by March 2023 (Figure 3).

### Adapting recruitment strategies to in-person environments

Upon relaxation of safety precautions in February 2022, our research staff was allowed onsite in two of the three CHCs as noted above. We worked with these clinics on protocols and began in-person recruitment at the first site in February 2022 and at the second site in May 2022. In-person recruitment enabled the team to reduce demands on the clinicians by explaining the study directly to participants and become a direct point of contact for the clinic staff. They were also able to describe the study to potential participants in more detail than clinicians could, establish a personalized rapport with participants, answer participant questions in real time, and in some cases, screen and consent participants onsite. This transition required modifications to IRB processes and study scripts. RAs needed flexibility to have more personalized approaches to building rapport with families but also to adapt to each CHC’s day-to-day routines to work unobtrusively alongside their staffs.

### Describing the study in culturally appropriate language

**Figure 4.**
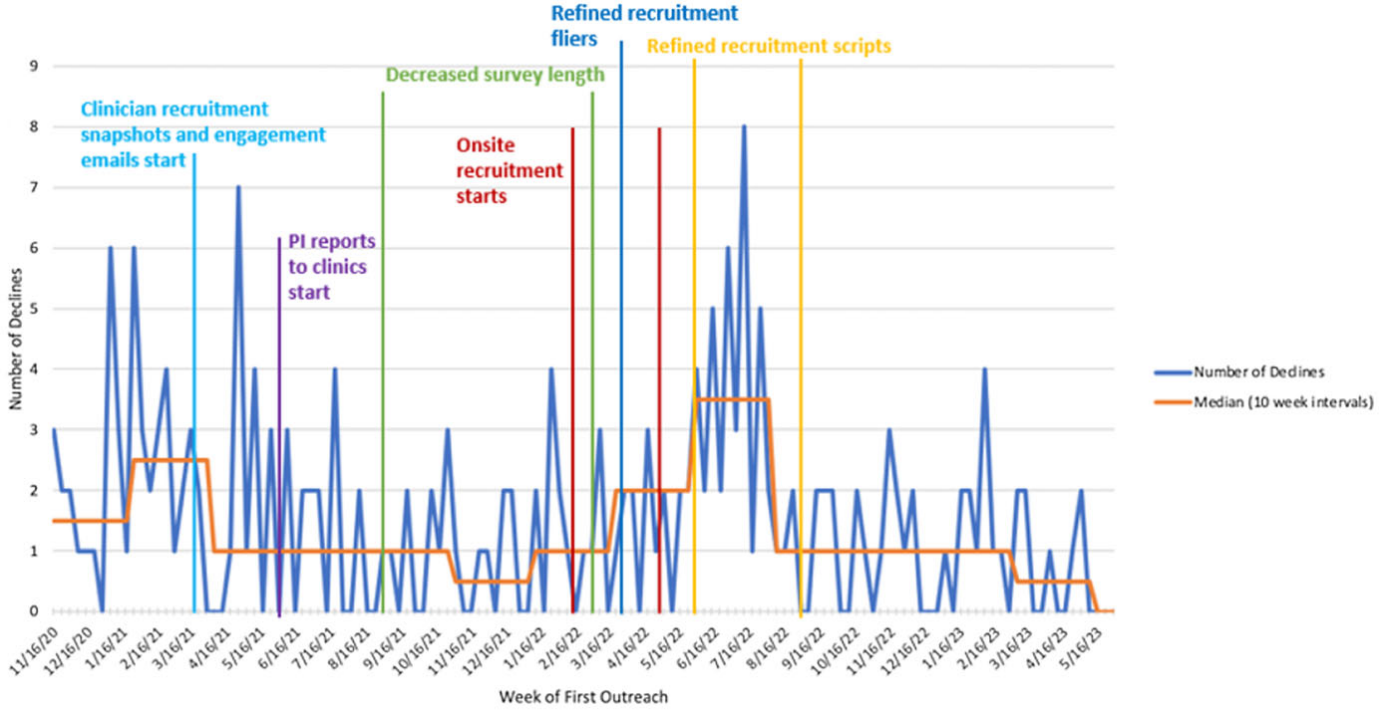
Total weekly declines.

Figure 2 illustrates the increase in referrals corresponding with in-person recruitment. However, with the increased number of referrals, we saw a corresponding increase in the number of individuals who declined participation (Figure 4).

In a collaborative manner, the RAs reflected on their interactions with participants with the core and larger team to identify potential reasons for an increase in the decline rate. The team reviewed and refined scripts and materials to enhance cultural and linguistic responsiveness. The introduction script was modified multiple times to eliminate unnecessary information, improve the English to Spanish translation, and clarify terminology that many parents misinterpreted. For example, many thought the word “estudio” (study) referred to a class they would need to take, or that the phrase “estudio de investigación” (research) implied biomedical procedures, like having blood drawn. Because the team could not remove these terms in accordance with IRB protocols, the team instead developed language to better explain the purpose of the research project and what would be expected of participants. The team modified the recruitment flyer multiple times during the recruitment period to address recurring questions like these, and, importantly, to add visual elements and timelines to help prospective participants understand participation expectations. The flyer was posted in patient exam rooms throughout the three CHCs, with extra copies available to hand out to patients.

## Discussion

This article describes how we applied quality improvement models, analytic approaches, and change strategies and tools to facilitate clinical trial participation among Latino parent–infant dyads from under-resourced communities. First, quality improvement models, such as PDSA cycles, provide opportunities for iterative cycles and dynamic strategies that respond to the unique challenges individuals from minoritized groups may face. Second, analytic approaches, such as time trend analyses (Figures 2–4), offer opportunities to create data-driven understandings of the problems and opportunities. Third, change strategies grounded in barriers understood to prevent the attainment of anticipated goals offer the opportunity to implement tailored and personalized approaches to address those barriers.

Although there has been some improvement in the involvement of people from minoritized groups in research, there is still a long way to go. The rapidly evolving COVID-19 pandemic conditions exacerbated participation barriers for people from minoritized groups and intensified the need for responsive recruitment strategies, flexibility, and frequent adaptation of procedures. Clinical and Translational Science Institutes (CTSI) and other investigators can build on this work to enhance participation among people from minoritized groups in research. Identifying recruitment challenges and workshopping ways of addressing them through PDSA cycles, cause and effect diagrams, run charts, and other QI tools can be adopted by researchers in other settings. Our experiences illustrate how quality improvement approaches like the Model for Improvement can enhance the conduct of clinical and translational science and such strategies can be adapted to local context. Our experiences also support the critical need to attend to culture, language, and literacy in developing and implementing recruitment strategies and study protocols.

The application of quality improvement models, strategies, and tools in this study adds to an emerging body of work that points toward their potential in enhancing how investigators and institutions conduct clinical translational science. Despite being widely applied in healthcare settings, their use in clinical translational science has not kept up with other fields and industries. Other CTSI’s have developed innovative training programs to encourage their uptake [26]. Other investigators have used quality improvement strategies to enhance participation and retention in longitudinal surveys [27]. The underrepresentation of people from minoritized groups in clinical trials is gaining attention as an urgent issue that threatens the external validity of treatment science and the uptake of innovations into everyday practice [15]. Applying quality improvement strategies to achieve this goal warrants additional testing.

Trust building and research staff with whom potential participants can identify have long been viewed as critical to building trust with people from minoritized groups to engage in research. Our team had cultivated relationships with community organizations and students who became our staff for many years, which facilitated trust and partnership building with the community in which we were working. Even with attention to these key concepts, we found that ensuring cultural, linguistic, and literacy adaptation was critical to promote participation. These findings correspond with work by Bell and colleagues [28] who leveraged best practices in multicultural and multilingual research to increase enrollment among people from minoritized groups. Further work is needed to augment the participation of community members to maximize relevance including strategies like codesign of materials [29].

### Limitations

This work is subject to certain limitations. First, we focused solely on Latino parent–infant dyads given our research questions and because they have been historically marginalized and underrepresented in research. However, our findings may not generalize to individuals from other groups or people at other stages of the life course. Second, the study occurred at three CHCs, and additional work is needed to apply quality improvement strategies across large-scale research studies across multiple sites. Third, other QI tools such as statistical process control chart methods could enhance rigor. Fourth, transcreation rather than simple translation would offer an approach to engage community members in the production of tailored materials. This is an important direction for future work. Lastly, we incorporated some changes simultaneously and thus could not estimate the independent effects of each change despite seeing an improvement in recruitment.

## Conclusion

Despite these limitations, we found that applying quality improvement concepts, strategies, and tools enhanced our recruitment efforts and helped to ensure clinical trial participation. We found that the use of strategies such as intentional team formation, participatory approaches to setting goals, establishing measures, selecting changes, and small iterative tests of change and implementation of changes were highly practical and helped our team overcome several barriers, even in the context of the COVID-19 pandemic. Other investigators and CTSIs may leverage such approaches to enhance participation in clinical trials and promote equity in clinical translational science.
